# Towards optimized tissue regeneration: a new 3D printable bioink of alginate/cellulose hydrogel loaded with thrombocyte concentrate

**DOI:** 10.3389/fbioe.2024.1363380

**Published:** 2024-03-26

**Authors:** Till Grandjean, Natarajan Perumal, Caroline Manicam, Björn Matthey, Tao Wu, Daniel G. E. Thiem, Stefan Stein, Dirk Henrich, Peer W. Kämmerer, Bilal Al-Nawas, Ulrike Ritz, Sebastian Blatt

**Affiliations:** ^1^ Department of Orthopedics and Traumatology, University Medical Center of the Johannes Gutenberg University Mainz, Mainz, Germany; ^2^ Department of Ophthalmology, University Medical Center of the Johannes Gutenberg University Mainz, Mainz, Germany; ^3^ Fraunhofer Institute for Ceramic Technologies and Systems (Fraunhofer IKTS), Dresden, Germany; ^4^ Department of Oral and Maxillofacial Surgery, University Medical Center of the Johannes Gutenberg University Mainz, Mainz, Germany; ^5^ Georg-Speyer-Haus, Institute for Tumor Biology and Experimental Therapy, Frankfurt am Main, Germany; ^6^ Department of Trauma, Hand and Reconstructive Surgery, University Hospital, Goethe University Frankfurt, Frankfurt am Main, Germany; ^7^ Platform for Biomaterial Research, BiomaTiCS Group, University Medical Center of the Johannes Gutenberg University Mainz, Mainz, Germany

**Keywords:** additive manufacturing, bioprinting, platelet rich fibrin, hydrogel, reconstruction

## Abstract

**Introduction:**

Autologous platelet concentrate (APC) are pro-angiogenic and can promote wound healing and tissue repair, also in combination with other biomaterials. However, challenging defect situations remain demanding. 3D bioprinting of an APC based bioink encapsulated in a hydrogel could overcome this limitation with enhanced physio-mechanical interface, growth factor retention/secretion and defect-personalized shape to ultimately enhance regeneration.

**Methods:**

This study used extrusion-based bioprinting to create a novel bioink of alginate/cellulose hydrogel loaded with thrombocyte concentrate. Chemico-physical testing exhibited an amorphous structure characterized by high shape fidelity. Cytotoxicity assay and incubation of human osteogenic sarcoma cells (SaOs2) exposed excellent biocompatibility. enzyme-linked immunosorbent assay analysis confirmed pro-angiogenic growth factor release of the printed constructs, and co-incubation with HUVECS displayed proper cell viability and proliferation. Chorioallantoic membrane (CAM) assay explored the pro-angiogenic potential of the prints *in vivo*. Detailed proteome and secretome analysis revealed a substantial amount and homologous presence of pro-angiogenic proteins in the 3D construct.

**Results:**

This study demonstrated a 3D bioprinting approach to fabricate a novel bioink of alginate/cellulose hydrogel loaded with thrombocyte concentrate with high shape fidelity, biocompatibility, and substantial pro-angiogenic properties.

**Conclusion:**

This approach may be suitable for challenging physiological and anatomical defect situations when translated into clinical use.

## Introduction

Reconstructive craniomaxillofacial-surgery addresses defect situations in the anatomical head and neck region due to trauma, cancer and other pathologies or infections (Khan, 2008; Amrollahi et al., 2016; Eguia et al., 2020). These defects remain clinical challenging with possible implications for functional (mastication, swallowing, phonation) and aesthetic outcome of the patients that directly affect quality of life. Autologous free flap transfer is the current gold standard in reconstruction, but can be hampered by patient’s morbidity (Tatullo et al., 2012; Turnbull et al., 2018). Here, biomaterials of different origin and classes are in need to replace restricted autologous tissue transfer. As an innovative drug delivery system, the class of autologous platelet concentrate (APC) can promote wound healing and tissue repair. Therefore, it is heavily used in regenerative and reconstructive surgery such as orthopedic procedures, cardiac surgery, plastic surgery, gynecology, urology, and medical esthetics as well as craniomaxillofacial-surgery (Gupta et al., 2021; Puricelli et al., 2023). The different cells involved, like thrombocytes and leukocytes confined in the structure-given fibrin net of these blood-derived products, release growth factors that can further interact with other cell types to initiate angiogenesis and stimulate immunological as well as other cellular processes involved in tissue regeneration (Miron et al., 2017a; Trzeciak et al., 2022). As a great advantage, the chair-side production and simple processing are timesaving and cost-efficient. Especially, second generation APC such as Platelet Rich Fibrin (PRF) that only need one centrifugation process and no addition of exogenous substances have gained increasing awareness (Kobayashi et al., 2016). On the downside, the multiple preparation protocols lead to different concentrate formulations that are partly controversial and discussed in terms of substantial variation of cell composition (e.g., with or without the inclusion of leukocytes) and growth factor release kinetics (e.g., outburst vs. slow and steady). These specifications of each formulation were subject to multiple previous studies (Dohan Ehrenfest et al., 2012; Schär et al., 2015; Masuki et al., 2016; Zwittnig et al., 2022). Centrifuge characteristics and centrifugation protocols can significantly affect the parameters (Dohan Ehrenfest et al., 2018; Al-Maawi et al., 2022). Besides these technical reasons for variations in the APC compositions and biological activity, inter-individual differences must be considered. Andrade Aldana et al. found an impact of gender and peripheral blood parameters on cellular content and fibrin concentrations of an APC (Andrade Aldana et al., 2022). Evanson et al. described differences in growth factor release depending on gender and age (Evanson et al., 2014). Cigarette smoke and uncontrolled diabetes mellitus type 2 affect platelet activation of APCs negatively (Srirangarajan et al., 2021; Das and Amaranath, 2022). In addition, and focusing on the biophysical aspect, APC show poor mechanical strength and rapid degradation that limit its usage in challenging defect situations (Mu et al., 2020). Our working group and others have attempted to solve these problems by combining APC with other biomaterials, such as collagen matrices (Blatt et al., 2020) or bone substitute materials (Blatt et al., 2021a; Blatt et al., 2021b). 3D bioprinting of a hydrogel loaded with APC could result in a direct physio-mechanical interface and enhance growth factor retention and prolonged and/or increased secretion. Secondly, defect situations could be addressed with personalized shape of the gel to support host integration. This could ultimately enhance regeneration.

Generally, 3D printing, also known as additive manufacturing (AM) or rapid prototyping, is defined as a versatile layer-by-layer fabrication technology of 3D objects through computer-aided design/computer-aided manufacturing (CAD/CAM). This technology has had an increasing impact on society and healthcare since its first introduction in the late 1980s (Ligon et al., 2017). Several process methods have been established, such as material extrusion, material jetting, binder jetting sheet lamination, vat photopolymerization, powder bed fusion, or directed energy deposition (Ligon et al., 2017). With substantial progress during the last decade, recent advances have enabled the 3D printing of biocompatible materials, cells, and supporting components into complex 3D functional living tissues (bioprinting). It shows excellent versatility in printing various biologics, including cells, tissue spheroids and strands, cell pellets, decellularized matrix components, micro-carriers, and cell-laden hydrogels. Therefore, 3D bioprinting in regenerative medicine is interesting in addressing the need for tissues and organs suitable for transplantation. It has already been used for the generation and transplantation of several tissues, including multilayered skin, bone, vascular grafts, tracheal splints, heart tissue, and cartilaginous structures (Murphy and Atala, 2014; Ozbolat and Hospodiuk, 2016).

Using inkjet (jet-based, drop-on-demand), microextrusion, or laser-assisted printing, a printable formulation with living cells (“cell/bioink”) can be encapsulated within polymeric hydrogels. These provide a suitable environment for cell growth and are highly customizable, allowing for several biochemical and biophysical properties to control cell functions, including cell adhesion, migration, proliferation, and differentiation, thus making them a valuable conduit for the fabrication of tissue constructs (Dell et al., 2022).

Goal of this study is to develop a novel bioink of alginate/cellulose hydrogel loaded with thrombocyte concentrate using extrusion based bioprinting to create a material inducing tissue regeneration in challenging defect situation where autologous flap transfer is not possible. Although other studies have been described employing these techniques or materials alone, to our knowledge the combination has not been described. Alginate is considered bioinert and cost-effective with a well-known gelling capacity but lacks the ability to support cell adhesion and spreading (Liu et al., 2023). The incorporation of APC in the formulation may overcome this limitation. Additionally, methylcellulose is a suitable material to enhance viscoelastic properties of the hydrogel (Ahlfeld et al., 2020; Banda Sánchez et al., 2023). The constructs were analyzed for their chemico-physical properties and influence on cell viability and proliferation *in vitro*. Additionally, their pro-angiogenic potential was assessed *in vivo*. Lastly, an extensive analysis of the total proteome and secretome of the prints was performed. This provides a fundamental understanding of the printed structures, aiding in comprehending future host interactions in clinical use.

## Materials and methods

### Preparation of injectable PRF (iPRF)

Injectable PRF (iPRF) from three healthy donors was manufactured as described in the literature (Miron et al., 2017b; Blatt et al., 2020). Briefly, after venous blood collection of 60 mL with unique vacutainer systems (i-PRF; Process for PRF, Nice, France), centrifugation of the blood was done according to the manufacturer’s protocol (700 rpm for 3 min, relative centrifugal force (rcf-max) = 60 *g* at 40° rotor angulation with a radius of 88 mm at the clot and 110 mm at the max; Duo Quattro centrifuge, Process for PRF, Nice, France). The iPRF for the preparation of the bioink was used as subsequently described. The study was conducted according to the guidelines of the Declaration of Helsinki and approved by the Ethics Committee of Landesärztekammer Rhineland-Palatine (no. 2019-14705_1, approved 09 September 2020). Informed consent was obtained from all subjects involved in the study.

### Establishment of the bioink

The elemental composition of the hydrogel, comprising sodium alginate and methylcellulose, along with their concentrations, is detailed elsewhere (Ahlfeld et al., 2020). In brief, sodium alginate powder was dissolved at a concentration of 3% (w/v) in phosphate-buffered saline (PBS) or injectable Platelet Rich Fibrin (i-PRF). Methylcellulose at a concentration of 9% (w/v) was then added to the sodium alginate solution, and the final hydrogel was allowed to swell for 20 min.

### Print process

The BioX from Cellink (Gothenburg, Sweden) was employed for 3D bioprinting of the hydrogel scaffolds. Printing was conducted at room temperature using a pneumatic printhead, a 3 mL pressure cartridge, and a conical nozzle with an inner diameter of 0.25 mm. The model template was created with the accessible computer-aided design (CAD) program FreeCAD. A simple cylinder with a diameter of 10 mm and a height of 3 mm was designed and provided to BioX as a.stl-file. The printer’s internal slicing program defined the internal structure of the model and the printing parameters, with a chosen 20% honeycomb structure for infill. The models were printed at a speed of 10–12 mm/s and a pressure of 70–80 kPa, using 24-well plates as the printing surface. The prepared hydrogel models were cross-linked with a 100 mM calcium chloride solution for 10 min.

### Scanning electron microscopy

For scanning electron microscopy (SEM), samples were fixed in a buffered formaldehyde solution. Following fixation, the samples underwent dehydration in ethanol, freeze-dried, sputtered with gold in an argon atmosphere. Subsequently, the samples were visualized using a scanning electron microscope (SEM; ESEM XL-30, Philips, Eindhoven, Netherlands).

### Histological evaluation

For histological evaluation of the hydrogels, hematoxylin and eosin (H&E) staining were performed following fixation in a buffered formaldehyde solution (4%) and subsequent paraffin embedding. Sections of 7 µm thickness were deparaffinized in xylene and rehydrated in ethanol with decreasing concentration. H&E staining was carried out using hematoxylin (Sigma-Aldrich, St. Louis, Missouri, United States) and eosin (Sigma-Aldrich) after rinsing with distilled water. Subsequently, staining was followed by dehydration with ethanol at increasing concentrations and treatment in xylene. Histological sections were photographed using a Leica MS 5 tripod (Leica Microsystems, Germany) and a JVC KY-F75U C-mount digital camera (JVC, Yokohama, Japan).

### X-ray diffraction and IR-spectroscopy

The X-ray diffraction (XRD) characterization was performed using an ID3003 TT (GE Sensing&Inspection technologies) with Cu K-alpha radiation and a position-sensitive detector (PSD). Solid samples were measured in the range of 5°–90°2θ with a step width of 0.03°2θ. For IR-spectroscopy, a Nicolet™ iS™ 50 FT-IR-spectrophotometer was employed with a resolution of 8 cm^-1^ and an average of 52 scans.

### Cytotoxicity assay with L929 cells

For *in vitro* cytotoxicity tests, L929 cells (Mouse fibroblast cell line, Thermo Fisher Scientific, Germany) were used in an assay according to DIN EN ISO 10993-5. The preparation and printing of the hydrogel were performed as described above. Ten thousand cells/well were seeded in a 96-well plate for 24 h. Subsequently, the L929 medium was replaced with supernatants from hydrogel models, each printed 24 h and 72 h before and then incubated for 24 h and 72 h, respectively. Following incubation, the treatment fluid was exchanged with 100 µL/well of a 1 mg/mL 3-(4,5- dimethylthiazol-2-yl)-2,5-diphenyltetrazolium bromide (MTT) (Sigma-Aldrich, St. Louis, MO, United States) solution in serum-free medium. The treated L929 cells were incubated with the MTT solution for 2 h before the solution was replaced by 100 µL isopropanol/well (Thermo Fisher Scientific, Waltham, MA, United States). Finally, absorbance was measured with the GloMax Multidetection (570/560 nm; Promega, Walldorf, Germany).

### Biocompatibility assay with SaOS-2 loaded i-PRF hydrogel scaffolds

To assess the biocompatibility of the i-PRF bioink, human osteogenic sarcoma cells (SaOS-2) were utilized. These cells were cultured as a monolayer at 37°C and 5% CO_2_ in Dulbecco’s Modified Eagle Medium F12 (DMEM F12) (Gibco, Life Technologies, Carlsbad, United States) supplemented with 10% (v/v) fetal calf serum (FCS) (Biochrom, Berlin, Germany) and 1% (v/v) penicillin-streptomycin (PS) (Sigma-Aldrich, Steinheim, Germany). The i-PRF bioink was prepared as described, and 100 µL of a SaOS-2 cell suspension was added per mL of bioink, resulting in a final cell concentration of 1.6×10^6^ cells/mL bioink. Cell-loaded hydrogels were then printed as previously described and incubated in DMEM F12 supplemented with 10% (v/v) FCS and 1% (v/v) PS at 37°C and 5% CO2. The viability of SaOS-2 cells entrapped in the hydrogel scaffolds was assessed 24 h and 96 h after printing using the alamarBlue^®^ assay (Life Technologies, Karlsruhe, Germany). The assay was performed and modified according to the manufacturer’s instructions. For each experiment, three cell-loaded models were covered with 500 µL of a 10% alamarBlue solution each for 5 h. The GloMax Multidetection System measured the fluorescence intensity (excitation 525 nm; emission 580–649 nm; Promega, Walldorf, Germany).

### VEGF, TGF-ß and PDGF ELISA

The growth factor expression of the printed constructs was analyzed by enzyme-linked immunosorbent assay (ELISA), according to the manufacturer’s protocol as previously described (Blatt et al., 2020). Samples were diluted 1:1 with Reagent Diluent. Antibodies for Vascular Endothelial Growth Factor (VEGF), Transforming Growth Factor ß1 (TGF-ß), and Platelet-Derived Growth Factor (PDGF) were employed following the manufacturer’s protocol (R&D Systems, Minneapolis, MN, United States). Absorbance at 450 nm was analyzed using an ELISA plate reader and specific software (SoftMax Pro 5.4, Molecular Devices, San Jose, CA, United States) at both the starting point (0 h) and after 72 h

### Chorioallantoic membrane (CAM) assay

The chorioallantoic membrane (CAM) assay was used to evaluate the influence of the prints (alginate/cellulose hydrogel with PBS vs. alginate/cellulose hydrogel with PRF bioink) on angiogenesis *in vivo*. As previously described (Blatt et al., 2020), fertilized white Leghorn chicken eggs (LSL Rhein-Main, Dieburg, Germany) were incubated at 38°C at constant humidity until the fourth day of embryological development. After removing 8–10 mL of egg white, a 3 × 3 cm^2^ window was cut into the eggshell under sterile conditions. On day 7, the samples were placed and incubated for an additional 96 h. Microscopic analysis at 50-fold magnification was then performed by entering the sample and defining the region of interest (ROI) using a digital microscope and its software (KEYENCE, Neu-Isenburg, Germany). Vessels were quantified using a deep learning application, as previously described (IKOSA, CAM assay [V3.10], ©KML Vision GmbH) (Annese et al., 2023). Subsequently, the embryos were euthanized by cutting the main vessels.

### Protein extraction for proteome analysis

The proteome analysis included the native Platelet Rich Fibrin (PRF_nat, *n* = 5), the bioink of alginate/cellulose hydrogel loaded with injectable Platelet Rich Fibrin (i-PRF) (PRF_3D, *n* = 5), and the secretome of the bioink of alginate/cellulose hydrogel loaded with i-PRF in PBS for 24 h at 4°C (PRF_sec, *n* = 5). Initially, the protein-extraction procedure for the respective samples was carried out using our in-house protocol (Perumal et al., 2019; Perumal et al., 2020). The samples were homogenized in the T-PER Tissue Protein Extraction Reagent (Thermo Scientific Inc., Waltham, MA, United States), and stainless-steel beads using a Bullet Blender homogenizer (BBY24M Bullet Blender Storm, Next Advance Inc., Averill Park, NY, United States). The supernatant of the tissue homogenates was subjected to buffer exchange and sample cleaning using 3 kDa centrifugal cut-off filters (Amicon Ultra 0.5 mL, Merck Millipore, Carrigtwohill, Ireland). Protein concentration estimation was determined by employing the bicinchoninic acid (BCA) protein assay kit (Pierce, Rockford, IL, United States) before in-solution trypsin digestion and peptide purification with SOLAµ™ SPE HRP plates (Thermo Fisher Scientific, Rockford, United States) according to the manufacturer’s instructions. The resulting peptide eluate was concentrated to dryness in a centrifugal vacuum evaporator and dissolved in 0.1% formic acid with the final protein concentration of 250 ng/μL.

### Mass spectrometry (MS)-based clinical proteomics analysis

The nano-liquid chromatography (nLC)-MS system utilized consisted of an EASY-nLC 1,200 system (Thermo Scientific, Rockford, United States) with an Acclaim PepMap RSLC, 75 µm × 50 cm, nanoViper analytical column (Thermo Scientific, Rockford, United States) directly coupled to ESI-LTQ-Orbitrap-XL MS (Thermo Scientific, Bremen, Germany), as described elsewhere (Perumal et al., 2022). Two microliters of each sample (500 ng) were used to fractionate peptides at a flow of 300 μL/min. Solvent A was LC-MS grade water with 0.1% (*v/v*) formic acid, and solvent B consisted of LC-MS grade acetonitrile with 20% (*v/v*) water and 0.1% (*v/v*) formic acid. The gradient per sample had a total run time of 240 min: 0–210 min: 5%–30% B, 210–220 min: 30%–100% B, 220–240 min: 100% B. In brief, the LTQ-Orbitrap operated in a data-dependent mode of acquisition, and survey full-scan MS spectra from m/z 300 to 2000 were acquired in the Orbitrap with a resolution of 30,000 at m/z 400 and a target automatic gain control (AGC) setting of 1.0 × 10^6^ ions. The ten most intense precursor ions were sequentially isolated for fragmentation and recorded in the LTQ (Perumal et al., 2022).

### Label-free quantitative proteomics analysis, functional annotation, and pathways analyses

The acquired continuum MS spectra were analyzed utilizing MaxQuant version 2.2.0.0 software (Cox and Mann, 2008; Luber et al., 2010; Cox et al., 2014; Tyanova et al., 2016). The tandem MS spectra were searched against the *Homo sapiens* database [Uniprot, reviewed (Swiss-Prot); Accession date, 14 January 2023; Annotated proteins, 20,404] with a target-decoy-based FDR for peptide and protein identification was set to 0.01. The summary of MaxQuant parameters employed in the current analysis is tabulated in Supplementary Data_01. The generated protein list from the MaxQuant analysis was used for subsequent statistical analysis with Perseus version 2.0.7.0 software (Tyanova et al., 2016). First, a log2 transformation of all protein intensities was done, and results were filtered to only include proteins with 100% valid measured values in at least one of the study groups. Missing values were imputed from a normal distribution in standard settings (width: 0.2, downshift: 1.8), enabling statistical analysis (Tyanova et al., 2016). The list of the significantly differentially abundant proteins in the designated comparison groups was used for functional annotation analysis employing the Ingenuity Pathway Analysis (IPA) (Krämer et al., 2014).

### Cell viability and proliferation of human umbilical vein endothelial cells (HUVECs)

Human umbilical vein endothelial cells (HUVECs) (PromoCell, Heidelberg, Germany) were cultured following the supplier’s recommendations, as previously described (Brendel et al., 2012). Lentiviral vectors encoding the enhanced green fluorescent protein were used for cell transduction to enable subsequent fluorescence microscopic examination of the cells on the printed hydrogel models. Viral supernatants, collected and concentrated from transfected 293T producer cells, were prepared as described earlier (Brendel et al., 2012). For gene transfer, 15,000 HUVECs were seeded into 24-well tissue culture plates (Greiner, Frickenhausen, Germany) in 500 μL media supplemented with 5 μg/mL protamine sulfate. Two rounds of transduction on days 1 and 3 were performed at a cumulative multiplicity of infection (MOI) of ∼100 to achieve >98% gene marking. After completion of the hydrogel models, 100 µL of a HUVECs cell suspension (5 × 10^6^ cells/mL) was pipetted onto each model. After 30 min, 1 mL of culture media was added to each model and then incubated at 37°C and 5% CO_2_. AlamarBlue assays were conducted at 24 h and 96 h after printing the models to assess cell viability and cell proliferation. The assay was performed and modified according to the manufacturer’s instructions. For each experiment, the hydrogel models were covered with 500 µL of a 10% alamarBlue solution each for 5 h. The GloMax Multidetection System measured the fluorescence intensity (excitation 525 nm; emission 580–649 nm). Three models were printed per experiment and covered with HUVECs. The experiment was repeated three times.

### Statistical analysis

Data were analyzed by GraphPad Prism (GraphPad Software, version 8.0.0 for Windows, San Diego, CA, United States, www.graphpad.com, accessed on 1 October 2022). The results are presented as mean ± standard deviation (SD). Statistical significance was determined through one-way ANOVA, with asterisks denoting significance levels as follows: *p < 0.05, **p ≤ 0.01, ***p ≤ 0.001, ****p ≤ 0.0001, and not significant (n.s., *p* > 0.05). Student’s two-sided t-test was employed for group comparisons, with *p* < 0.05 considered statistically significant to identify differentially abundant proteins in the label-free quantitative proteomics analysis. Unsupervised hierarchical clustering analysis of the identified differentially abundant proteins was performed according to Euclidean distance (Linkage, Average; Preprocess with k-means, enabled; Number of clusters, 300; Maximal number of iterations, 10; Number of restarts, 1). The top enriched terms of the gene ontology cellular component (GOCC), molecular types, biological functions upstream regulators, and canonical pathways of the differentially abundant proteins were presented with *p*-value calculated using Benjamini–Hochberg (B-H) multiple testing correction (one-sided Fisher’s exact test, -log B-H *p*-value >1.3).

## Results

### Alginate/cellulose hydrogel loaded with thrombocyte concentrate exhibits an amorphous structure characterized by high shape fidelity

**FIGURE 1:**
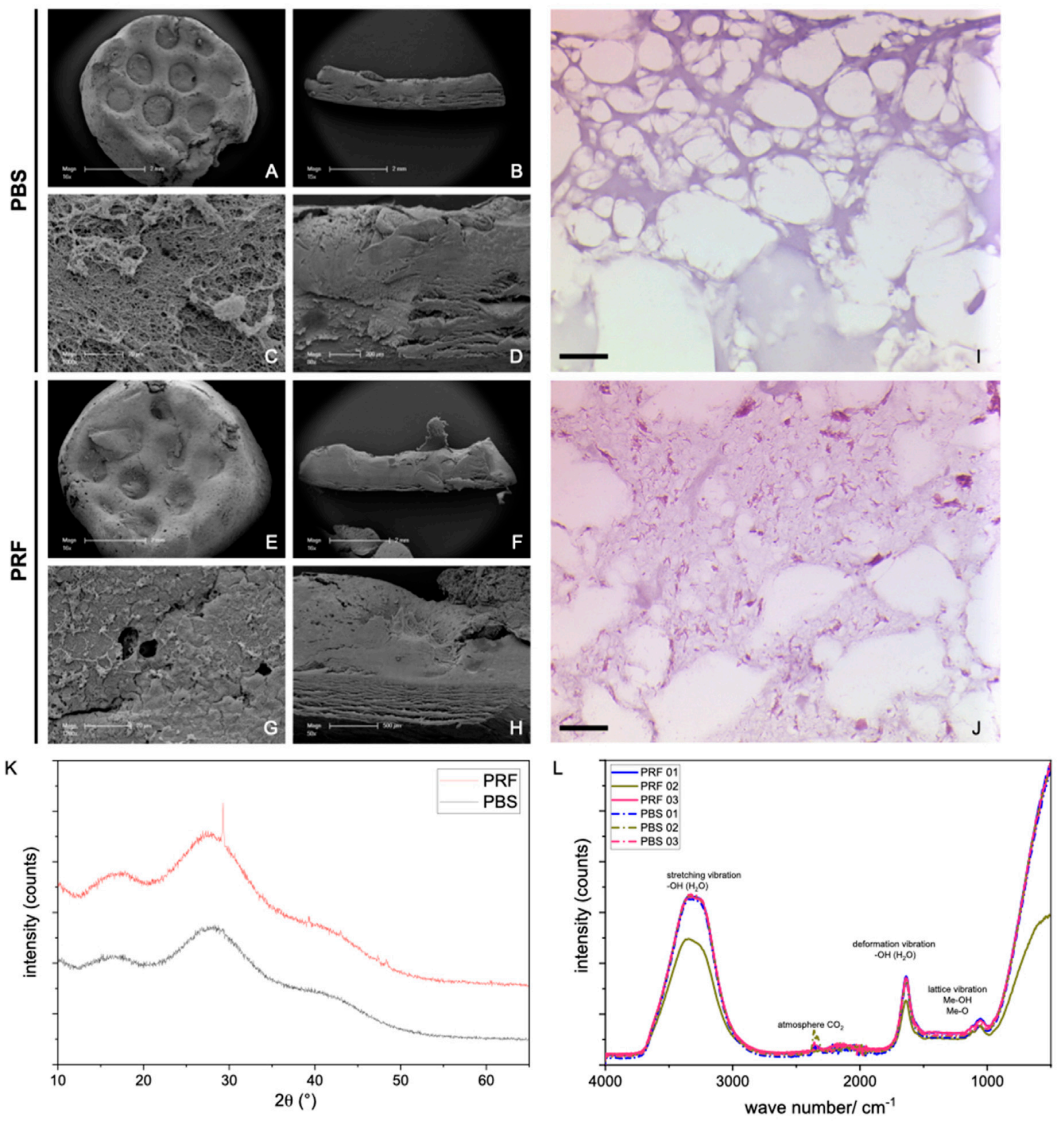
SEM images of 3D prints with alginate/cellulose hydrogel with PBS **(A–D)** vs. alginate/cellulose hydrogel loaded with iPRF **(E–H)**. Representative images of the surface in low **(A, E)** and high **(C, G)** magnification, as well as sections in low **(B and F)** and high **(D, H)** magnification, are shown. Furthermore, histological images of 3D prints with PBS **(I)** and loaded with PRF **(J)** after hematoxylin and eosin staining (Bar = 50 µm). XRD Analysis **(K)**: For both groups, broad, amorphous humps around 15°–35°2q represent a nearly full amorphous character of the analyzed samples (grey line: PBS samples, red line: PRF loaded constructs). In the PRF-loaded sample, various peaks appear at 29.3, 39.2, and 47.2°–48.7°2q representing the semi-crystalline character of the bioink itself. **(L)** In addition to intense water bands, there may be metal hydroxide vibrations or OH-group vibrations associated with polymers shown in the IR spectra (*n* = 3) for each sample, dashed line: PBS samples, solid line: PRF-loaded constructs).

Firstly, the surface characteristics of the prints were evaluated with scanning electron microscopy (SEM), while chemico-physical properties were assessed with X-ray diffraction (XRD) and IR-spectroscopy. SEM images of the hydrogel surfaces (Figure 1) revealed a smooth surface in lower magnification for both PBS (Figure 1A) and PRF-loaded hydrogels (Figure 1E). At higher magnification, small globular structures (approximately 0.5 µm in diameter) with long processes were observed on the surface of PRF-loaded prints (Figure 1G), absent on the surface of PBS control prints (Figure 1C). These structures likely represent the remnants of thrombocytes. Sections of the hydrogels demonstrated an internal network, with both hydrogels comprising dense lamellar structures and interposed open areas (Figures 1B, D, F, H). Subsequent histological evaluation of the hydrogels (Figures 1I/J) revealed a mesh of amorphic fibers in both PBS (Figure 1I) and PRF (Figure 1J). In PRF-loaded constructs, aggregates and fibers of high eosinophilic material were visible, which were absent in PBS native hydrogel prints.

The XRD analysis (Figure 1K) reveals the nearly complete amorphous nature of the samples, evident from the background and broad, amorphous humps observed around 15°–35°2θ. Notably, in the PRF-loaded constructs, distinct peaks emerge at 29.3, 39.2, and 47.2°–48.7°2θ. While these peaks could not be unequivocally assigned, they do not correspond to either alginate or cellulose. It is, therefore, plausible that they signify the semi-crystalline character intrinsic to the bioink itself. Additionally, the IR spectra, aside from featuring intense water bands, may exhibit vibrations associated with metal hydroxide or OH-groups in the polymers (Figure 1L).

### Alginate/cellulose hydrogel loaded with thrombocyte concentrate reduces cytotoxicity, increases cell viability and proliferation, and demonstrate substantial pro-angiogenic growth factor release

**FIGURE 2:**
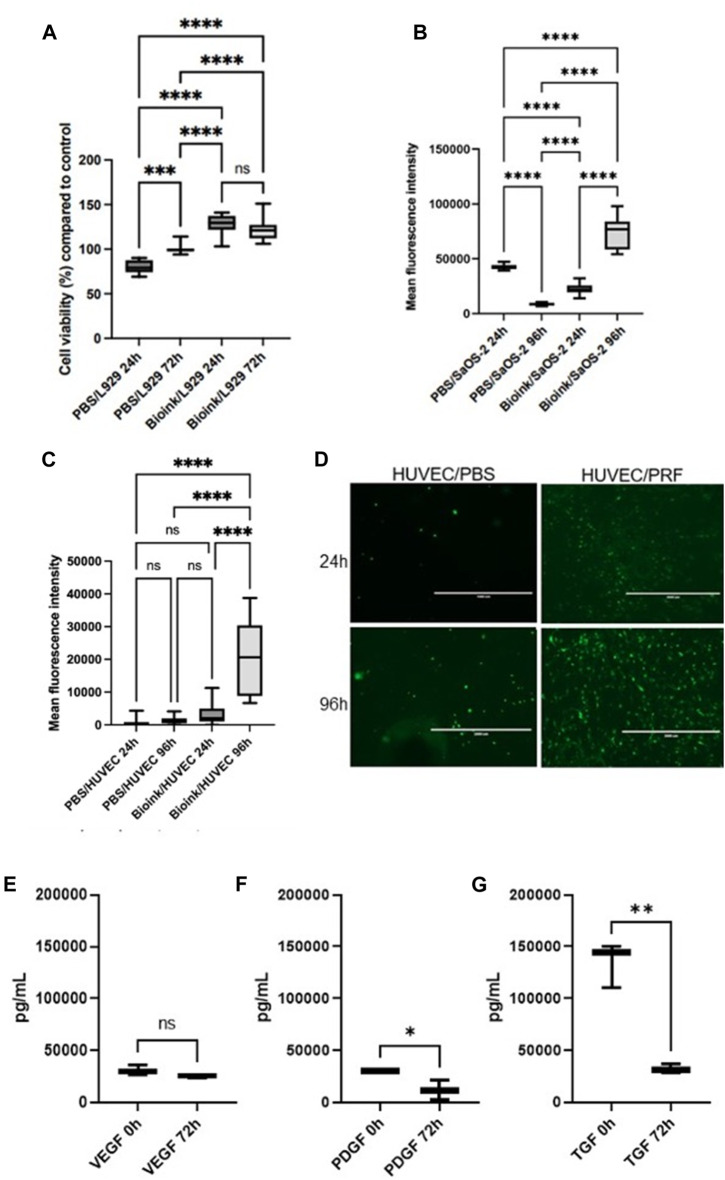
**(A)** Box plots illustrate the cell viability of L929 cells in the cytotoxicity assays. (according to DIN EN ISO 10993-5). No cytotoxic effects could be observed (as defined by ISO 10993-5 and indicated by a reduction in cell viability of >30% compared to the control). **(B)** Box plots show high cell viability after 24 h and increased viability after 96 h of incorporated SaOS-2 cells in the PRF-based bioink loaded alginate/cellulose hydrogels via AlamarBlue assay. **(C)** Box plots demonstrate cell viability and proliferation via Alamarblue assays of the HUVEC-seeded prints. **(D)** Representative fluorescence microscopic examinations of the GFP transduced HUVECs on the respective samples (PBS hydrogel models left, PRF incorporated prints right) after 24h and 96 h. Boxplot demonstrate growth factor expression of VEGF **(E)**, PDGF **(F)**, and TGF-ß **(G)** via ELISA of the respective growth factors of the alginate/cellulose hydrogel loaded with PRF bioink (pg/ml, *n* = 3, means ± SD, *p < 0.05, **p < 0.01, and ns determined by one-way ANOVA).

To assess potential cytotoxic effects, cytotoxicity assays were conducted using L929 cells, a standard cell line for such experiments following DIN EN ISO 10993-5 guidelines. The cells were exposed to medium supernatants derived from the hydrogel models previously incubated for 24 h and 72 h. No cytotoxic effects, as defined by ISO 10993-5 (indicated by a reduction in cell viability of >30% compared to the control), were observed with the supernatants from both the native PBS and PRF hydrogel models (Figure 2A). In contrast to PBS prints, the PRF hydrogel models exhibited a positive impact on the cell viability of L929 cells (>100% compared to the control). To assess the cell compatibility of the hydrogel, SaOS-2 cells were introduced to examine viability and proliferation after 24 h and 96 h using the AlamarBlue assay. Viability of the included SaOS-2 cells was demonstrated 24 h after the printing of the cell-loaded PRF hydrogel. Subsequent assays after 96 h revealed an increase in cell viability (Figure 2B), indicating an elevated cell proliferation rate of the SaOS-2 cells.

To simulate the physiological attachment processes of cells to the hydrogel model, HUVECs were cultured on the printed surfaces. AlamarBlue assays were employed to investigate potential positive effects of PRF-loaded hydrogel on cell viability and proliferation, conducted after 24 h and 96 h on the cell-populated models. Additionally, fluorescence images of cells transduced with GFP on the model surface were captured (Figures 2C, D). On the native PBS prints, low viability rates were observed for HUVECs after both 24 h and 96 h. In contrast, higher viability rates were evident for HUVECs on the PRF models after 24 h, and this significantly increased after 96 h (Figure 2C). Fluorescence microscopic examination of HUVECs on the PBS hydrogel models revealed few healthy, adherent cells. In contrast, HUVECs on the PRF models exhibited optically healthy and adherent cells after 24 h. Moreover, after 72 h, significantly more cells were observed by fluorescence microscopy, aligning with the results of the AlamarBlue assays (Figure 2D).

In terms of growth factor expression, ELISA analyses of cell free models revealed substantial expression and release of the respective growth factors of the alginate/cellulose hydrogel loaded with PRF bioink. At the initial measurement point immediately after incubation (0 h), the analyzed pro-angiogenic growth factors VEGF (Figure 2E), PDGF (Figure 2F), and TGF-ß (Figure 2G) exhibited high expression levels. After 3 days (72 h) of incubation, the release of growth factors decreased but remained evident. Notably, VEGF expression remained consistently high without a significant decrease over time.

### The addition of the bioink to hydrogel constructs of alginate/cellulose enhances the pro-angiogenic potential in ovo

**FIGURE 3:**
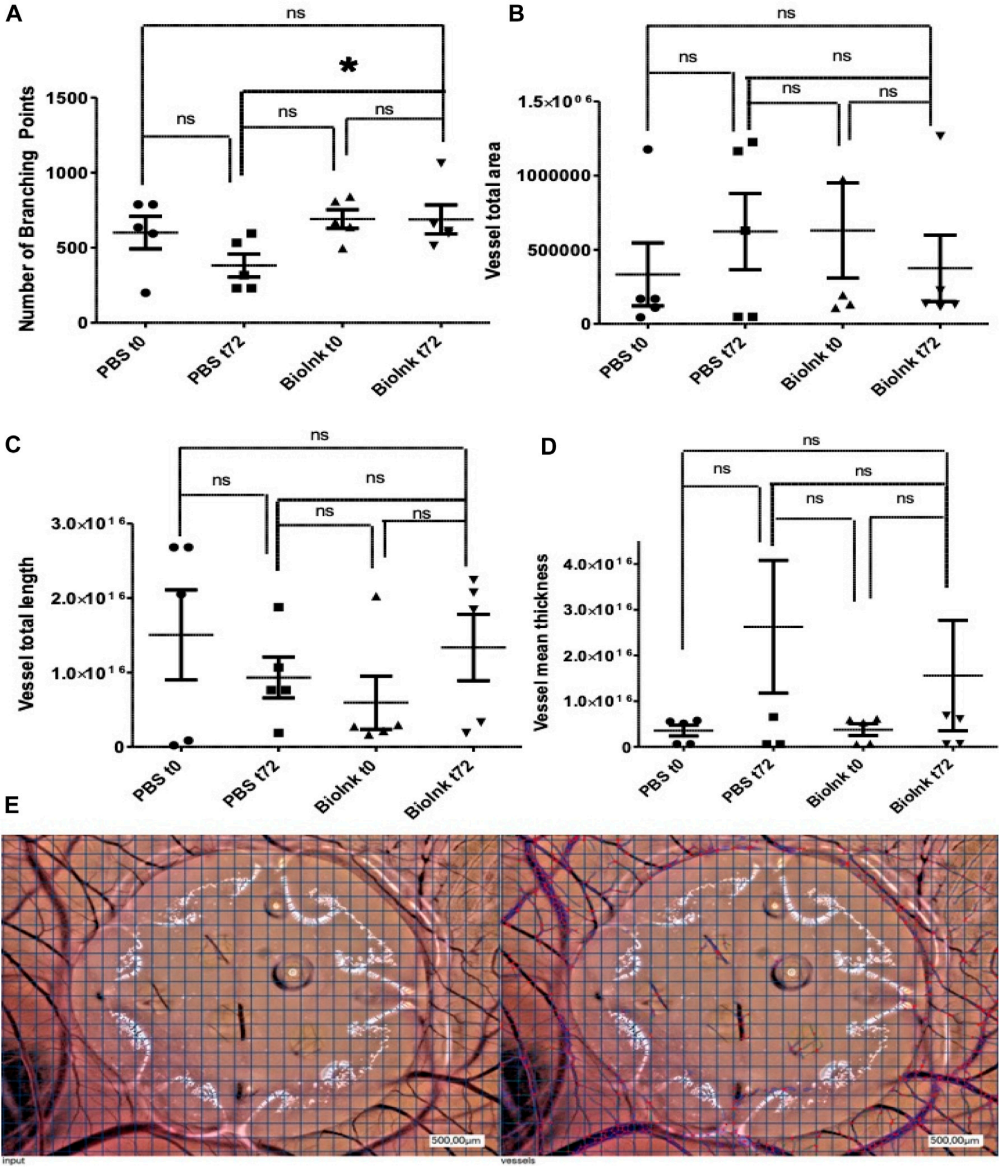
CAM assay to assess pro-angiogenic properties of the PBS and PRF prints *in vivo*: number of branching points **(A)** significantly enhanced in hydrogels loaded with PRF vs. PBS hydrogels after 72 h (t72). In contrast, the presence of the PRF loaded prints reduced mean vessel total area **(B)**, but increased total vessel length **(C)** and mean vessel thickness **(D)** without reaching statistical significance. Representative images **(E)** of the hydrogels on the CAM at 72 h (input, left side) and the automatic cell count via respective software (right side) (pg/ml, *n* = 5, means ± SD,*p < 0.05 and ns determined by one-way ANOVA).

To assess pro-angiogenic properties of the prints *in vivo*, the CAM assay was performed. This revealed a significantly enhanced number of branching points when comparing mean numbers between native PBS hydrogels and hydrogels loaded with PRF, incubated from 0h to 72 h. In contrast, native hydrogels tended to increase the mean vessel total area of the CAM, a trend that was reduced in the presence of PRF-loaded constructs, though without reaching statistical significance. Hydrogels loaded with PRF demonstrated a tendency to positively influence total vessel length and mean vessel thickness compared to PBS hydrogels (Figure 3).

### Proteome and secretome revealed a substantial amount and homologous distribution of pro-angiogenic proteins in the 3D-construct

**FIGURE 4:**
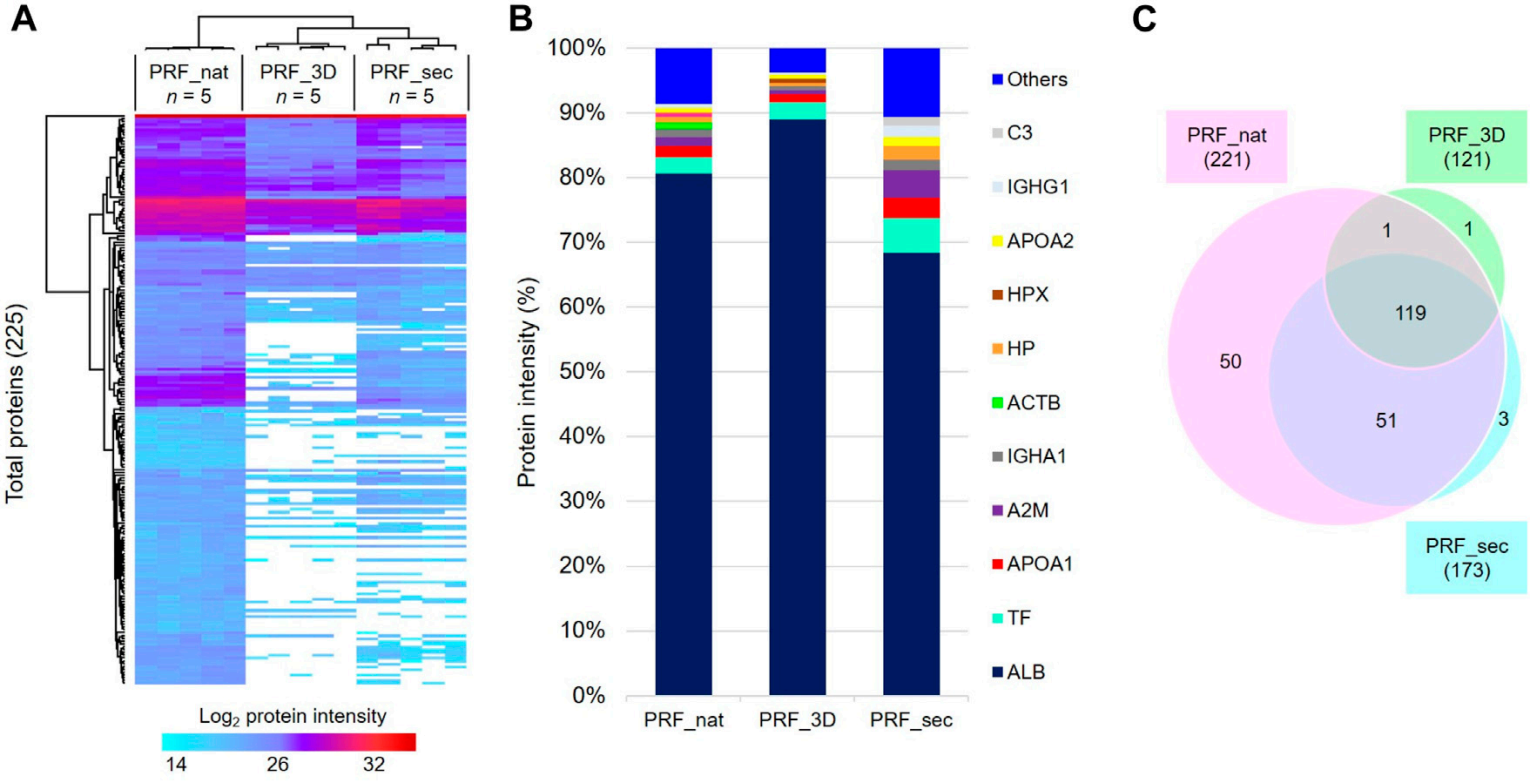
Proteome profiles of PRF in the native and 3D forms and the PRF secretome: **(A)** Heat map depicts the hierarchical clustering of all 225 total proteins based on the log2 protein intensity related to the designated groups. **(B)** Bar charts show the degree of mean percentage of top abundant proteins in the designated groups. **(C)** The venn diagram illustrates the total number of proteins identified in all samples after 24 h. PRF_nat: native platelet concentrates; PRF_3D: 3D-printed platelet concentrates; PRF_sec: 3D-PRF secretion/secretome.

Label-free proteomics analysis identified 225 proteins with a 1% false discovery rate (FDR) across all 5 biological replicates in the designated groups (Figure 4A; complete protein list in Supplementary Data S2). The top ten most abundant proteins, constituting approximately 90% of the total identified proteins, included serum albumin (ALB; ∼70%), serotransferrin (TF), apolipoprotein A1 (APOA1), alpha-2-macroglobulin (A2M), IGHA1, and beta-actin (ACTB) (Figure 4B).

PRF_nat exhibited the highest number of identified proteins (221), while PRF_3D had the lowest (121). PRF_sec, with 173 proteins, showed a higher number than PRF_3D. Notably, 119 proteins (∼53%) were common to all three samples, with 51 shared between PRF_nat and PRF_sec (Figure 4C). PRF_nat had the highest number of exclusively identified proteins (50, ∼22%).

**FIGURE 5:**
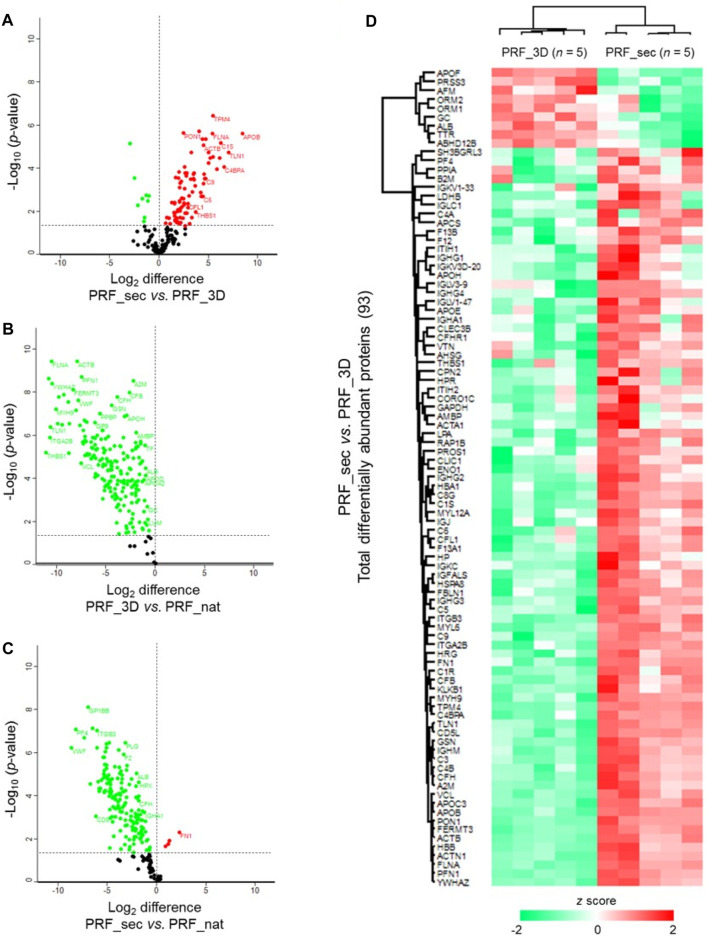
Differential expression profiles of PRF proteins: Volcano plots illustrate the significantly differentially abundant proteins identified based on the log2 difference in the **(A)** PRF_sec vs. PRF_3D, **(B)** PRF_3D vs. PRF_nat and **(C)** PRF_sec vs. PRF_nat. Significance threshold is at *p* < 0.05. **(D)** The heat map depicts the hierarchical clustering of the significantly differentially abundant proteins in the PRF_sec vs. PRF_3D. Upregulated proteins are shown in red, and the downregulated proteins are in green. PRF_nat: native platelet concentrates; PRF_3D: 3D-printed platelet concentrates; PRF_sec: 3D-PRF secretion.

Statistical analysis revealed 93 significantly differentially abundant proteins in PRF_sec vs. PRF_3D (Figure 5A; Supplementary Data S3). The majority (84 proteins, ∼90%) were increased in abundance in the PRF_3D secretome, including APOB, TLN1, C4BPA, C1S, YWHAZ, and ITGA2B (Figure 5D). Further functional annotation and pathways analyses indicated higher complexity and abundance of proteins in PRF_nat compared to PRF_3D (Figure 5B) and PRF_sec (Figure 5C; Supplementary Data S3).

**FIGURE 6:**
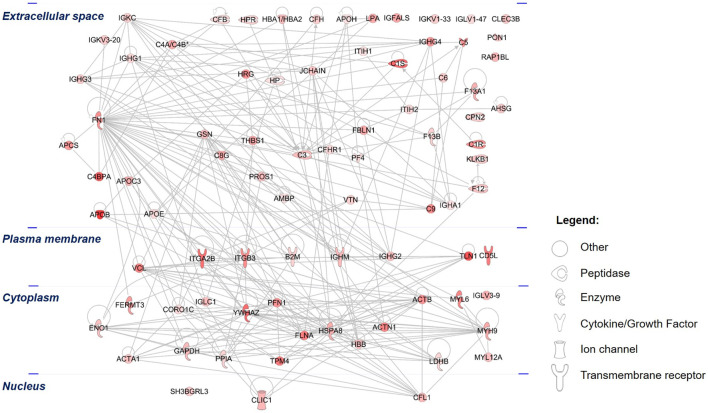
Protein-protein interactions (PPIs) of the differentially upregulated secretome proteins: The PPI of the PRF secretome shows the networks of differentially abundant proteins, which were highly increased in abundance in the secretome compared to the 3D PRF. The different color intensities correspond to the degree of differential expression. Proteins are annotated according to their cellular localization and are depicted as different shapes that represent the functional classes of the proteins.

In an effort to elucidate the functions of the 84 differentially upregulated PRF-secreted protein clusters, the IPA tool was utilized to analyze the most significant canonical pathways, diseases, and biological functions. The protein-protein interaction (PPI) network analysis revealed that the majority of the secretome comprised peptidases and transporter proteins localized in the extracellular space (Figure 6).

**FIGURE 7:**
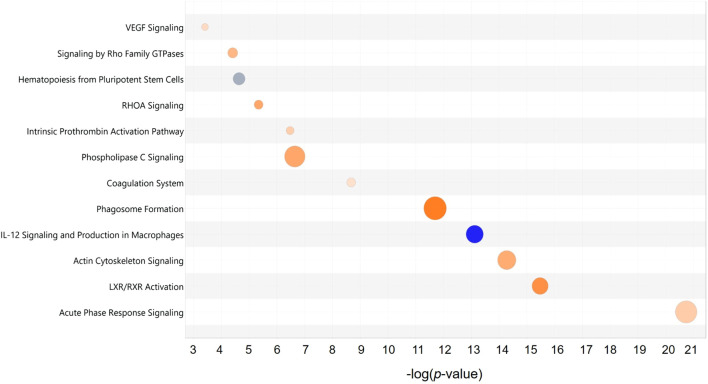
Top significant canonical pathways of the PRF secretome: Bubble plot of the top significantly (*p* < 0.001) enriched canonical pathways associated with the differentially abundant proteins in the PRF secretome. Overall z-scores are represented by the color orange, which indicates activation, blue indicates inhibition of the signaling pathways; and grey indicates not quantifiable activity pattern. The size and color of each bubble represent a number of differentially abundant proteins in each pathway and z-score, respectively.

Among these proteins, an extracellular protein, fibronectin (FN1), showed the highest number of PPIs, followed by two cytoplasmic proteins composed of YWHAZ and heat shock cognate 71 kDa protein (HSPA8). Further analysis demonstrated that the top significantly affected canonical pathways were involved mainly in acute phase response signaling (*p* = 2.00 × 10^−13^, z-score = 1.41), LXR/RXR activation (*p* = 3.16 × 10^−16^, z-score = 3.05), actin cytoskeleton signaling (*p* = 5.01 × 10^−15^, z-score = 2.31, coagulation system (*p* = 2.09 × 10^−9^, z-score = 0.82), signaling by Rho family GTPases (*p* = 3.89 × 10^−5^ z-score = 2.00), and VEGF signaling (*p* = 3.89 × 10^−4^, z-score = 1.00) (Figure 7). All these signaling pathways were found at high level in the PRF secretome except for the IL-12 signaling and production in macrophages, which was expressed at low level (*p* = 7.94 × 10^−14^, z-score = −3.74).

**FIGURE 8:**
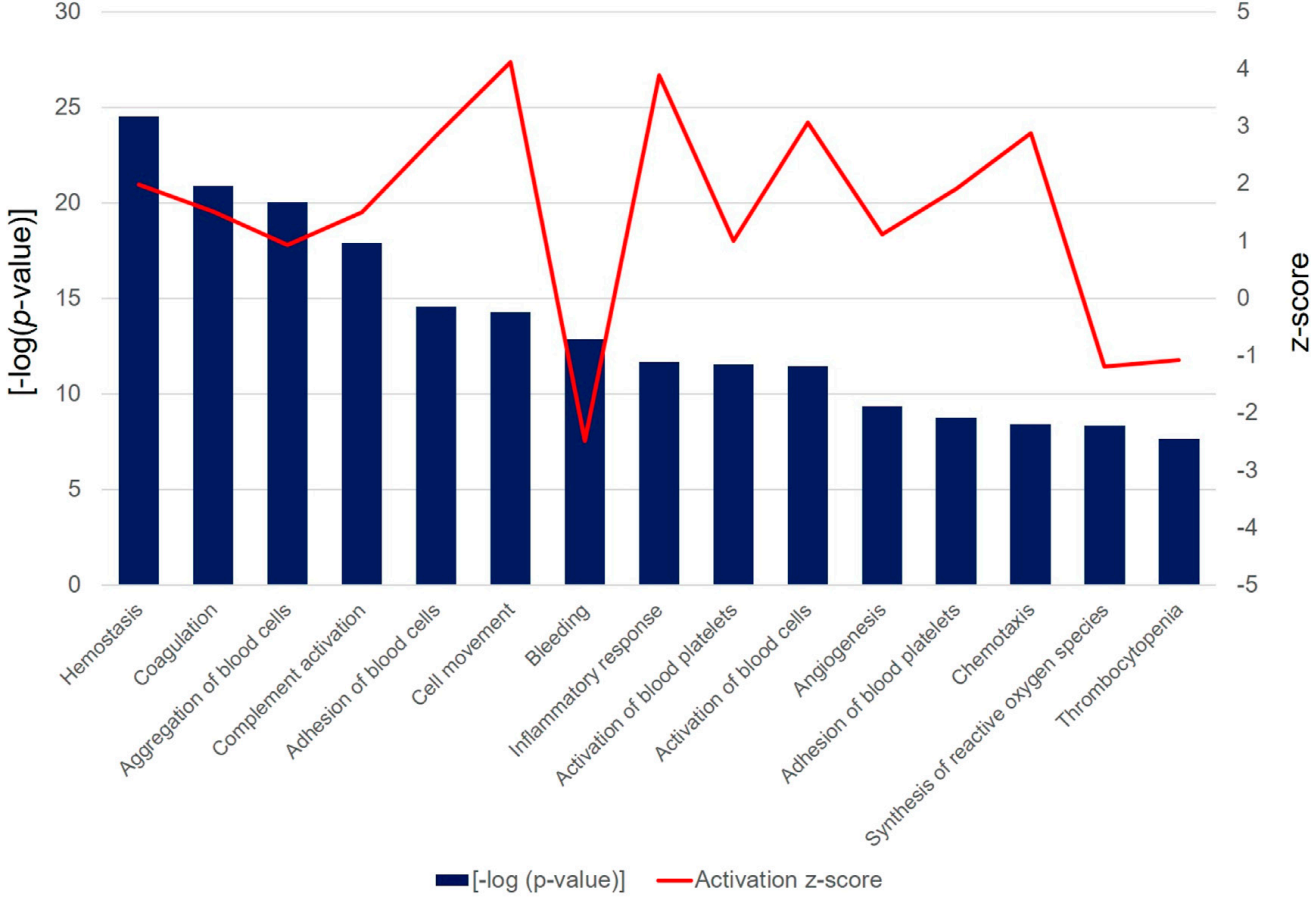
The bar chart depicts the top 15 significant [-log (*p*-value) > 1.3] biological functions attributed to the differentially abundant PRF secretome proteins. Bars represent the -log (*p*-value) (left y-axis) and the line represents the activation z-score (right y-axis) of each function and disease.

In the functional analysis of the PRF secretome, pathways associated with hemostasis (*p* = 3.04 × 10^−25^, z-score = 1.20), complement activation (*p* = 1.22 × 10^−18^, z-score = 1.50), adhesion of blood cells (*p* = 2.64 × 10^−15^, z-score = 2.84), cell movement (*p* = 5.45 × 10^−15^, z-score = 4.12), inflammatory response (*p* = 2.13 × 10^−12^, z-score = 3.9), activation of blood platelets (*p* = 2.90 × 10^−12^, z-score = 1.00), and angiogenesis (*p* = 4.15 × 10^−10^, z-score = 1.11) were expressed at high levels. Interestingly, the bleeding functionality (*p* = 1.34 × 10^−13^, z-score = −2.48), synthesis of reactive oxygen species (*p* = 4.59 × 10^−9^, z-score = −1.18), and thrombocytopenia (*p* = 2.08 × 10^−8^, z-score = −1.07) were found at low level (Figure 8).

**FIGURE 9:**
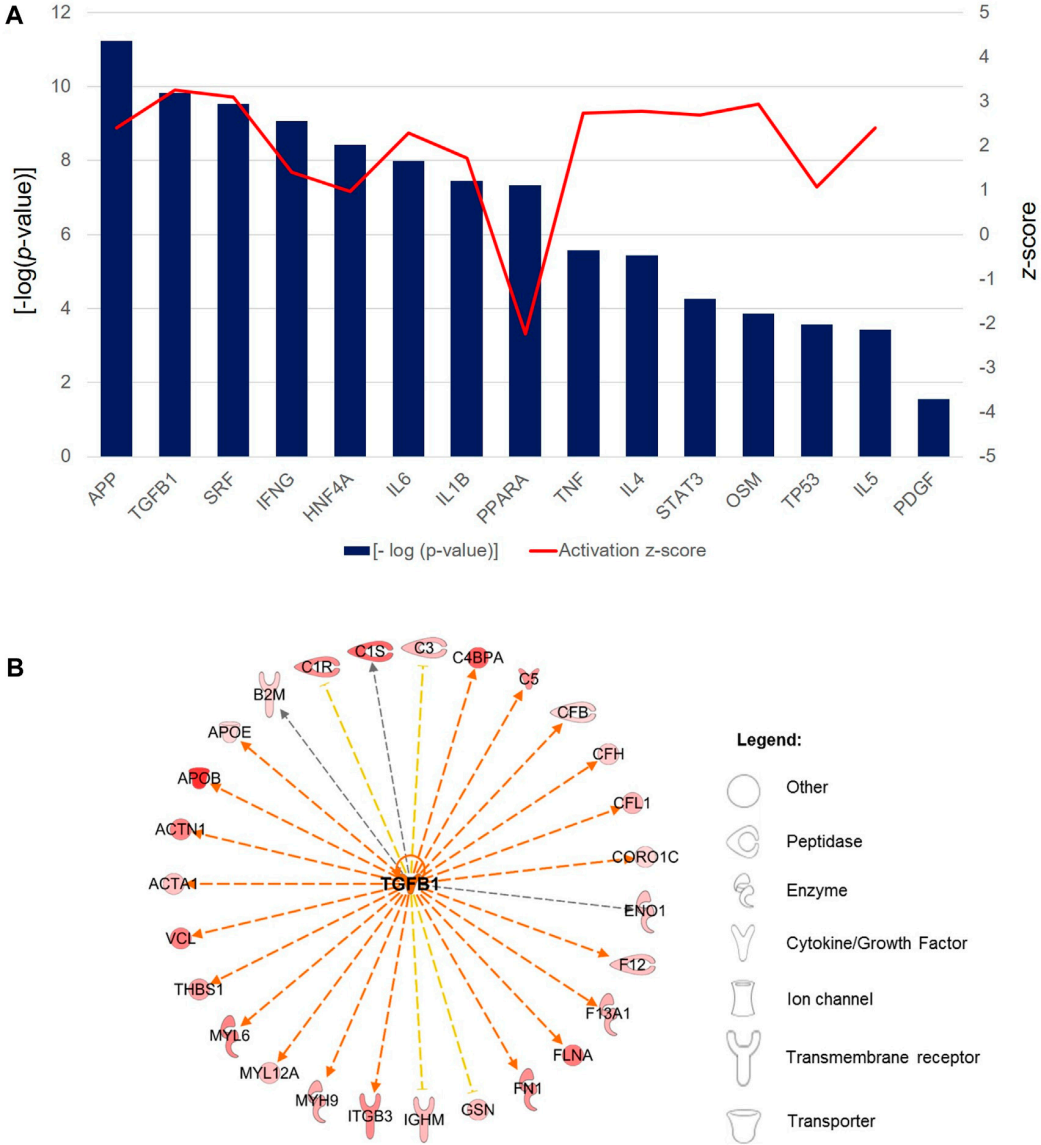
Predicted upstream regulators of the PRF secretome. **(A)** Bar chart shows the profiles of top selected significant [-log (*p*-value) > 1.3] upstream regulators associated with the significantly differentially abundant proteins in the PRF secretome. Bars represent the -log (*p*-value) (left y-axis) and the line represents the activation z-score (right y-axis) of each regulator. **(B)** Exemplary interaction network profile of TGF-β-regulated differentially expressed secreted proteins. Different shapes represent the various classes of proteins.

Finally, the role of upstream regulator molecules, including transcription factors and cytokines, which were predicted to significantly modulate the downstream expression of PRF secretome was investigated. The large majority of regulators consisted of cytokines composed of interferon-gamma (IFNG; *p* = 8.61 × 10^−10^, z-score = 1.40), interleukin-6 (IL-6; *p* = 1.03 × 10^−8^, z-score = 2.29), interleukin-1β (IL-1β; *p* = 3.53 × 10^−8^, z-score = 1.73), tumor necrosis factor (TNF; *p* = 2.67 × 10^−6^, z-score = 2.74), interleukin-4 (IL-4; *p* = 3.62 × 10^−6^, z-score = 2.78), oncostatin M (OSM; *p* = 1.40 × 10^−4^, z-score = 2.94) and interleukin-5 (IL-5; *p* = 3.69 × 10^−4^, z-score = 2.40) (Figure 9A). Another cluster of regulators comprised transcription regulators, namely, serum response factor (SRF; *p* = 2.91 × 10^−10^, z-score = 3.09), hepatocyte nuclear factor 4 alpha (HNF4A; *p* = 3.82 × 10^−9^, z-score = 0.97), signal transducer and activator of transcription 3 (STAT3; *p* = 5.48 × 10^−5^, z-score = 2.70) and tumor protein 53 (tp53; *p* = 2.75 × 10^−4^, z-score = 1.08). All regulators were predicted to be activated in the downstream regulation of PRF secretome apart from the ligand-dependent nuclear receptor, peroxisome proliferator-activated receptor alpha (PPARA; *p* = 4.63 × 10^−8^, z-score = −2.24), the activity of which was significantly inhibited. On the other hand, although the activity pattern of the platelet-derived growth factor (PDGF) was not quantifiable, the participation of this dimeric glycoprotein in the regulation of the PRF secretome was also significant (*p* = 2.79 × 10^−2^). Among these regulators, TGF-β is one of the most significantly implicated regulator with the highest activation z-score (*p* = 1.48 × 10^−10^, z-score = 3.26). The exemplary profile of the proteins governed by activated TGF-β showed the downstream regulation of a large cluster of enzymes, peptidases, and proteins with other molecular functions (Figure 9B).

## Discussion

The presented study focuses on the 3D extrusion-based bioprinting of a novel bioink consisting of an autologous platelet concentrate (PRF) incorporated into an alginate/cellulose hydrogel construct. Key findings include the successful feasibility of the bioprinting process and the demonstration of high biocompatibility. The chemico-physical properties with a high shape fidelity of the printed constructs are well suited for personalized regeneration concepts. ELISA analyses further reveal a significant release of pro-angiogenic growth factors from the printed constructs, contributing to accelerated cell viability and proliferation. Pro-angiogenic potential is partially confirmed *in vivo*. Notably, this study presents a comprehensive validation of the print’s proteome and secretome, indicating a substantial presence of abundant proteins both in the native PRF and the printed hydrogel loaded with PRF-based bioink. The significant overlap in proteins between the native and 3D-printed platelet concentrates and secretome underscores the homologous distribution of proteins in the 3D construct. This proves the feasibility of the print process with no degradation of the respective proteins.

In the context of bioprinting processes, bioinks are considered biologically functional products that incorporate living cells and biomaterials. These bioinks can be processed with cells as suspensions or dispersions, deposited simultaneously in a separate printing process, or used as transient support materials (Groll et al., 2018). Alginate, known for its low toxicity, gelling capacity, and cost-effectiveness, is a widely used material for bioink formulation. While alginate is cytocompatible and bioinert, it may lack the ability to support cell adhesion and spreading. To overcome this limitation, different proteins and bioactive factors are often incorporated into alginate-based bioinks to enhance their physicochemical and biological properties and promote cell adhesion, proliferation, and differentiation (Liu et al., 2023). In our approach, a 3% w/v alginate concentration was chosen based on its favorable outcomes in terms of cell viability (Banda Sánchez et al., 2023). To enhance viscoelastic properties of the hydrogel, alginate was blended with methyl cellulose (MC), a cut-compatible water-soluble cellulose derivative. A 9% w/v concentration of MC was used, following literature recommendations (Ahlfeld et al., 2020; Banda Sánchez et al., 2023). In recent years, bioinks containing human plasma have been introduced to strengthen the patient-specific concept (Ahlfeld et al., 2020). Ahlfeld et al. introduced a bioink based on fresh frozen plasma blended with alginate/methylcellulose, demonstrating that the inclusion of plasma promoted cell spreading and the formation of intercellular interactions in the hydrogel. The authors hypothesized that fibrinogen in plasma played a key role in enhancing cell attachment (Ahlfeld et al., 2020). In our approach, Platelet Rich Fibrin (PRF) was used instead of plasma, considering the 210-fold higher concentration of platelets and fibrin found in PRF compared to plasma. This substitution was anticipated to accelerate the regenerative potential of the bioinks. The analysis of the viability and adherence potential of the sarcoma cell line SaOS-2 presented in our study aligns with these assumptions.

A limitation of our study is the lack of investigation into the impact of native Platelet Rich Fibrin (PRF) and the bioink-containing print on other cell lines beyond SaOS-2, L929 cells, and HUVECs. Specifically, mineralizing cells related to bone tissue regeneration were not evaluated. Recent research has demonstrated the positive effects of PRF on cell proliferation, differentiation, mineralization, and inflammation reduction, especially in the context of bone tissue regeneration (de Lima Barbosa et al., 2023). Other 3D printing approaches incorporating PRF have utilized human osteoblast-like MG-63 cell lines and bone marrow-derived stem cells, showing higher viability (Song et al., 2018; Rastegar et al., 2021). It can be hypothesized that the presented approach may have similar effects on these cell lines. However, we focused on soft tissue healing, and the potential implications on bony regeneration were not addressed. Ongoing studies are dedicated to exploring these aspects.

Our results suggests that Platelet Rich Fibrin (PRF) does not counteract but rather supports the high viscoelastic properties of the hydrogel. In a study by Mu et al. (2020), biphasic hydrogels based on gelatin nanoparticles and injectable PRF (iPRF) were developed. These hydrogels were described as bioactive and adaptable to the topographical and mechanical conditions of irregular-shaped defects. The outstanding mechanical properties of these hydrogels were considered a valuable solution for addressing clinical challenges in treating bone defects with a high degree of local complexity. In alignment with this, we demonstrated that the printed constructs had a nearly complete amorphous character. Interestingly, the loading of the constructs with PRF resulted in a semi-crystalline character. This finding is noteworthy for future clinical applications where prints must be adapted to complex and irregular-shaped defect situations. The ability of PRF to influence the mechanical properties of the hydrogel may contribute to its utility in addressing specific challenges associated with irregular-shaped defects.

As shown in previous studies, a combination of different materials and iPRF [for example, the combination of collagen membranes or bone substitute materials with an autologous platelet concentrate (Blatt et al., 2020; Blatt et al. 2021a; Blatt et al. 2021b)] led to similar release kinetics in comparison to the native APC. Since APC are well known for their pro-angiogenic potential, this approach can be used for the prevascularization of the respective biomaterial and, therefore, enhancement of the regenerative potential (Blatt et al., 2021a). The interaction between the biomaterial and APC can be amplified through bioink formulation via 3D bioprinting. The blotting process in bioprinting establishes a direct physio-mechanical interface, potentially leading to growth factor retention and prolonged and/or increased secretion. Our results demonstrate growth factor release kinetics of the printed constructs comparable to the known release kinetics of native APC (Blatt et al., 2020). In particular, the stable expression of vascular endothelial growth factor (VEGF) at a constantly high level during the measurement suggests prolonged and sustained kinetics in bioink formulation via 3D bioprinting. VEGF is a key regulator in the process of neo blood vessel formation (Carmeliet, 2003; Carmeliet, 2005; Potente et al., 2011; Eelen et al., 2018). Principally, one can differ between vasculogenesis and angiogenesis. Whereas vasculogenesis describes the blood vessel formation *de novo* from circulating endothelial precursor cells and is mainly associated with embryological development (one of the exceptions are specific pathophysiological processes such as carcinogenesis), angiogenesis defines the neo vessel formation via sprouting and intussusception of pre-existing ones (Djonov et al., 2003; Asahara and Kawamoto, 2004). The presented release kinetics of the prints with low level of PDGF and TGF-ß and consistent VEGF level after 72 h can initially support this processes and underscore their pro-angiogenic potential. Additionally, it is known the APC can cause induction of genes associated with angiogenesis and wound healing such as Fibroblas-Growth-Factor 2 (FGF-2) or Angiopoetins that contribute to the discussed influence on angiogenesis (Singh et al., 2022). Since TGF-ß is the key upstream regulator of FGF2 (Gualandris et al., 2022), we focused on VEGF, PDGF and TGF-ß analysis (as essential regulator of angiogenesis) in this study.

However, a major limitation of the presented study is the lack of long-term analysis of specific growth factor retention and secretion. Other studies, such as the one by Yi et al. (2022), have used a bioink based on PRF and alginate/gelatin, showing prolonged and sustained growth factor release with the advantage of personalized molding. The extended release kinetics of APC-containing bioink in this study are hypothesized to result from the mechanical properties of the bioink polymer and electrostatic interactions between growth factors and the gelatin or fibrin network matrix (Yi et al., 2022). The presence of metal hydroxide vibrations or OH-group vibrations associated with polymers observed within the IR spectra supports the hypothesis of a direct physio-mechanical interface between the hydrogel and APC. This characteristic makes the approach ideal for reconstructive procedures of soft tissue, where regeneration typically requires 6–8 weeks.

The literature describes various autologous platelet concentrate (APC) preparation protocols for 3D bioprinting applications (Song et al., 2018; Bahraminasab et al., 2022; Le et al., 2023; Sui et al., 2023). APC preparations of different generations vary in manufacturing protocols, mechanical properties, and biological activity, leading to different growth factor release kinetics (Dohan Ehrenfest et al., 2012). These release kinetics appear to be preserved after the printing process. Studies, such as one by Le et al. (2023), have shown that hydrogels incorporated with PRF demonstrate higher growth factor release for PDGF and VEGF compared to first-generation APC-incorporated gels. The wide range of 3D printing methods, from filament-free printing to fused deposition modeling and extrusion-based printing, contributes to the heterogeneity in incorporating APC into bioinks (Song et al., 2018; Bahraminasab et al., 2022; Le et al., 2023; Ren et al., 2023). Currently, there is no study that directly compares these different protocols, making it challenging to recommend a single best method for incorporating APC into bioinks. However, *in-situ* bioprinting is highlighted as a promising method for individually addressing defect situations directly in patients. This approach involves incorporating autologous platelet concentrates, easily accessible and readily available at the bedside, into individually formed hydrogels intraoperatively. These hydrogels can then be directly printed into the defect for reconstruction purposes (Zhao et al., 2022).

In the present study, not only the specific growth factor release was measured, but the pro-angiogenic character of the constructs was also assessed *in vivo* with the CAM assay. The assay has several limitations such as a nonspecific inflammatory response and its high sensitivity to environmental factors (e.g., pH or osmolarity). Additionally, timing of the angiogenesis analyses is crucial. Especially the initial exponential embryological vessel growth and rearrangement of existing vessels makes it sometimes hard to distinguish from a falsely increased vascular density. On the other hand, it is a relatively simple, quick, and low-cost model to properly assess angiogenesis *in vivo*. Therefore, it seems superior to other *in vitro* methods such as tube formation assays (Ribatti, 2016). Recent studies demonstrated this assay feasible to investigate the influence of APC in combination with biomaterials on angiogenic potential (Blatt et al., 2020; Blatt et al. 2021a; Blatt et al. 2021b). To study these effects, different methods of quantifying vasculature have been used and described including the manual count of the vessels. However, this is quite time-consuming and prone to human bias whereas automated and semi-automated applications enable the quantitative assessment of various vasculature morphometric and spatial parameters (Faihs et al., 2022). With recent developments in artificial intelligence, new possibilities for image analysis have emerged. The IKOSA platform, as used in this study, facilitates AI-based analysis of vascular networks on CAM images, the underlying image analysis model is based on a fully convolutional neural network, which uses an InceptionResNetv2 as an encoder and a U-Net based structure as a decoder path (Faihs et al., 2022). The analysis results in a segmentation map in terms of a binary mask representing the vessel area at pixel level. The study by Faihs et al. (2022) suggests that the IKOSA method is considered the most precise for analyzing vascular parameters, specifically the branching points and mean vascular thickness. This conclusion is based on a comparison with the standard manual count. Additionally, IKOSA is noted to provide an advantage by allowing the evaluation of further vascular parameters that cannot be effectively assessed through manual counting.

Our results suggest that the presence of autologous platelet concentrate (APC) enhanced vessel density in the CAM (chorioallantoic membrane) based on references to previous studies (Blatt et al., 2020; Blatt et al. 2021a; Blatt et al. 2022). Although the vessel density, reflected by the total area of the vessels and mean vessel thickness, did not show a significant increase for the constructs loaded with PRF-based bioink compared to native hydrogels, there was a marked tendency for an increased vessel total length and a significant enhancement in the number of branching points. These findings show a notable influence of the constructs loaded with the PRF-based bioink on angiogenesis, opening the possibility for further small animal studies before potential clinical translation of the technique.

As a significant finding, we present a thoroughly analysis of the total secretome of the native autologous platelet concentrate (APC) and the constructed bioink. This analysis provided insights into the presence of proteins in the 3D construct compared to the native PRF, demonstrating the feasibility of the printing approach. It is noteworthy that, so far, only two studies have investigated the proteome of PRF, and there is a lack of prior research analyzing the secretome of a PRF-based bioink. The focus of the analysis was primarily on the influence on angiogenesis, considering that APCs are best known for their pro-angiogenic potential, emphasizing their clinical relevance in reconstructive surgery (Miron et al., 2017c). While angiogenesis is crucial for tissue regeneration, it is important to note that other regenerative activities also play a role in addressing challenging defects, and further investigations may be warranted to explore a broader spectrum of biological functions related to regeneration.

Hermida-Nogueira et al. (2020) performed Liquid chromatography–mass spectrometry (LC-MS/MS) and longitudinal analysis by Sequential Window Acquisition of all Theoretical Mass Spectra (SWATH). They identified a total of 705 proteins within the PRF as well as 202 differentially secreted proteins over time. In their approach, Yaprak et al. (2018) used 2D gel electrophoresis. Here, the matrix-assisted laser desorption/ionization coupled to time of flight mass spectrometry (MALDI-TOF/TOF) analysis revealed contrary results with few abundant proteins. The discrepancy between the mentioned study results could be explained by the PRF extraction method used by Yaprak et al. (2018) to exclude fibrin. This may have led to the removal of other proteins along with the fibrin that explains the low level of protein identifications. The proteomics analysis conducted in this study provided evidence of the presence of many proteins identified in the native PRF. This finding further supports the high biological activity of APC, such as PRF, and underscores their clinical utility in reconstructive surgery. Additionally, the 3D-printed hydrogels loaded with PRF exhibited a reduced but similar number of proteins compared to the native PRF. This suggests that the process of 3D printing and incorporation into hydrogels did not significantly alter the overall protein composition, highlighting the stability of the bioink and its potential for use in regenerative applications.

In contrast, the secretome of the 3D constructs incorporating Platelet Rich Fibrin (PRF) revealed the identification of more proteins compared to the native APC. Although approximately 22% of the proteins were exclusively found in the native APC, around 53% of these proteins were common to all three sample types (native PRF, 3D-PRF, and secretome). Our study represents the first detailed exploration of the biological activity of the new 3D printable bioink, composed of an alginate/cellulose hydrogel loaded with PRF. The findings include the identification of top abundant PRF proteins, such as ALB, A2M, APOA1, and complement C3 (C3), which mainly consist of platelet-released factors identified in previous investigations (Tiedemann et al., 2022).

As further key findings, proteomics validated high level of TGF-β as one of the essential upstream regulators, as well as VEGF and PDGF. Serum response factor was the second-highest activated upstream regulator. SRF is a known downstream mediator of VEGF signaling in endothelial cells and a critical requirement for VEGF-induced angiogenesis by starting endothelial cell migration and proliferation (Chai et al., 2004). Correspondingly, the downstream proteins regulated by SRF comprised mostly cytoskeletal proteins (ACTA1, ACTB, ACTN1, CFL1, FLNA, ITGA2B, MYH9, TLN1, and VCL) with several similar proteins also annotated in VEGF signaling (ACTA1, ACTB, ACTN1, and VCL). Therefore SRF is seen as a master regulator of the actin cytoskeleton and contractile apparatus (Miano et al., 2007). VEGF promotes SRF expression and nuclear translocation and increases SRF binding activity to DNA in endothelial cells through both Rho-actin and MEK-ERK dependent signaling pathways (Chai et al., 2004). Therefore, SRF seems crucial for the known clinical regenerative potential of APC and should be investigated in future studies. Next, Peroxisome proliferator-activated receptor alpha (PPARα), an upstream regulator, was expressed at low levels. Platelet PPARa critically mediates platelet activation and contributes to the prothrombotic status under hyperlipidemia (Li et al., 2022). PPARα and PPARγ were identified to mediate anti-angiogenic processes that limit platelet activation and thrombosis (Wagner and Wagner, 2020; Li et al., 2022). Again, this emphasizes the pro-angiogenic potential of PRF and its 3D-printed constructs presented in this study.

Furthermore, influence of the prints on immune cells and respective pathways that trigger an immune response are critical and decide between integration and rejection of the foreign material in the host. In particular, the role of TGF-β is emphasized as a key cytokine that regulates various aspects of T cell biology, including development, activation, proliferation, differentiation, and apoptosis (Chen, 2023). The subpopulation of FOXP3+ regulatory T cells (Treg) is identified as crucial for maintaining immune homeostasis by suppressing the activation of potentially harmful effector T cells (Ou et al., 2023). Our results points out that TGF-β, a significant upstream regulator in PRF and its 3D-printed constructs, may induce the differentiation of Tregs. This differentiation process is associated with the Glycoprotein A repetitions predominant (GARP), a transmembrane protein and TGF-β binding partner. The regulation of immune capacity by inducing regulatory CD4^+^ T cells is attributed to GARP, as previously described in native PRF (Trzeciak et al., 2022). Furthermore, native PRF polarized macrophages to a “M0/M2-like” phenotype (Trzeciak et al., 2022). Given the significant overlap in proteome and secretome between native PRF and the 3D-printed constructs, our results suggest that the printed constructs may exhibit similar anti-inflammatory effects. However, it acknowledges the need for further preclinical trials to specifically demonstrate the anti-inflammatory capacity of the 3D-printed constructs with PRF.

Our study has several significant limitations. Firstly, the research is constrained to *in vitro* and in ovo analyses, and there is a lack of *in vivo* testing in actual wound healing scenarios. The challenges associated with animal research on autologous platelet concentrates (APC), particularly Platelet Rich Fibrin (PRF), are highlighted in other studies: obtaining high-quality APC, quick coagulation times, difficulties in clot activator use, and limitations in accessing appropriate vessels, especially in small animals like rats or rabbits, are noted as potential challenges for *in vivo* studies (Mourão et al., 2023). Our approach is positioned as a feasibility study demonstrating the printability, physico-mechanical properties, and biological activities of a hydrogel with a bioink containing thrombocyte concentrate. To adhere to the “3R concept” (Replacement, Reduction, Refinement) of animal welfare, the study primarily focuses on *in vitro* and in ovo analyses. Future animal research is suggested to address the challenges in APC research in animals and to evaluate the long-term effects and stability of the bioink in living organisms. Another limitation is the analysis of cell viability, proliferation, and migration, which was conducted with a limited number of cell lines. We focused on cells involved in soft tissue healing, and the implications for osteogenic differentiations of stem cells were not evaluated. Future studies should consider a broader range of cell types, especially those relevant to bone tissue regeneration. Furthermore, no long-term analysis of growth factor retention and secretion of the prints was conducted. Long-term evaluations in future (animal) studies are suggested to assess the sustained effects for improved tissue regeneration using the proposed approach. Lastly, scalability and changes in bioink’s properties after up-scale to large defect situations remain unexploited. This is a challenge in 3D bioprinting research that most studies of the current literature lacks. Printing of intricate scaffold architectures can help to design hierarchical formulations and structures (Agrawal and Hussain, 2023) that optimizes scalability and can lead to new *in situ* print applications (Zhao et al., 2022).

In summary, while our study provides valuable insights into the feasibility and initial biological activities of the proposed 3D-printed constructs, it underscores the importance of addressing these limitations in subsequent research, including *in vivo* studies, long-term analyses and scalability.

For the outlook, 4D printing holds enormous potential when additive manufacturing is included in tissue regeneration procedures in biomedical and healthcare settings. The goal of this approach is to incorporate time as the fourth dimension, providing the prints with the flexibility to modify their morphology with the help of smart materials. In short, 3D printed materials can be transformed into 4D prints via an external stimulus such as temperature or radiation, enabling them to function as smart dynamic structures. With a second external impulse, transformation takes place (Agarwal et al., 2023). With this new technique, drug delivery systems can be encapsulated into the 3D construct and released upon a specific stimulus. For the presented approach, this method is conceivable for APC in cooperated bioinks and subsequent growth factor release on the defect site, for example, in wound healing approaches due to pH changes.

## Conclusion

A new 3D bioprinting approach to create an alginate/cellulose hydrogel loaded with a thrombocyte concentrate bioink with excellent rheological characteristics and high biocompatibility was demonstrated. The printed hydrogel loaded with Platelet Rich Fibrin (PRF) revealed the presence of numerous abundant proteins. The substantial overlap in proteins between native and 3D-printed platelet concentrates, as well as their secretomes, indicates the homologous presence of proteins in the 3D construct, thus demonstrating the feasibility of the 3D printing process without degradation of the respective protein. This finding provides evidence for its pro-angiogenic potential and underscores its suitability and functionality in clinical settings.

## Data Availability

The mass spectrometry proteomics data presented in the study are deposited to the ProteomeXchange Consortium via the PRIDE (Perez-Riverol et al., 2022) partner repository with the dataset identifier PXD050629.
